# Changes to Physical Activity, Sitting Time, Eating Behaviours and Barriers to Exercise during the First COVID-19 ‘Lockdown’ in an English Cohort

**DOI:** 10.3390/ijerph181910025

**Published:** 2021-09-24

**Authors:** Lindsy Kass, Terun Desai, Keith Sullivan, Daniel Muniz, Amy Wells

**Affiliations:** Life and Medical Science, University of Hertfordshire, Hertfordshire AL10 9EU, UK; t.desai@herts.ac.uk (T.D.); k.sullivan3@herts.ac.uk (K.S.); d.muniz@herts.ac.uk (D.M.); a.v.wells@herts.ac.uk (A.W.)

**Keywords:** COVID-19, behaviours change, physical activity, barriers to exercise, sitting time

## Abstract

This study aimed to determine the effect of the first English national COVID-19 lockdown on physical activity (PA), sitting time, eating behaviours and body mass in an adult cohort. This was further examined to determine whether conforming to recommended guidelines on PA and sedentary behaviour was improved. Based on an online survey (*n* = 818) incorporating the International Physical Activity Questionnaire Short Form (IPAQ-SF), self-reported body mass change showed that in 32.2% of participants body mass increased, with 39.1% reporting an increase in food intake. Never exercising at the gym or undertaking an exercise class (online or live), increased by 50.8% during lockdown, with 53.5% changing from exercising frequently to never exercising, suggesting a lack of engagement with online and home workouts. However, outdoor running and cycling >2 times/week increased by 38% during lockdown. Walking at least 30 min continuously on >2 occasions/week increased by 70% during lockdown with minimum 10-min walks on 7 days per week increasing by 23%. The lockdown had a negative impact on sitting time (>8 h a day), which increased by 43.6% on weekdays and 121% at weekends. Furthermore, sitting <4 h/day decreased during lockdown (46.5% and 25.6% for weekdays and weekends, respectively). Those citing tiredness or lack of time as a barrier to exercise reduced by 16% and 60%, respectively, from pre-lockdown to during lockdown. More of the sedentary group met the Public Health England PA recommendations, however most participants still did not meet the UK Government guidelines for PA. Improvements in health per additional minutes of physical activity will be proportionately greater in those previously doing <30 min/week, the area where most improvements were found although, conversely sitting time was greatly increased. This study may assist in informing whether future lifestyle changes could improve the health of the population.

## 1. Introduction

The World Health Organization (WHO) declared a Public Health Emergency of International Concern due to the outbreak caused by severe acute respiratory syndrome coronavirus 2 (SARS-CoV-2) on 30 January 2020, and a pandemic on 11 March 2020 [1]. To reduce the speed of the transmission of the SARS-CoV-2, several governments implemented measures to limit the movements of its citizens. The United Kingdom (UK) Government enforced a lockdown on the UK population from 23 March 2020, resulting in most adults and children staying at home, only allowed to leave the household for certain activities [2]. These measures were relaxed in mid-May 2020 and enforced again in November 2020.

During the first phase of lockdown (23 March to 13 May 2020) many people were furloughed or allowed to work from home, resulting in a change in living conditions, and lifestyle. The lockdown itself posed challenges for those working with school or nursery age children, as childcare was not available, except for children of key workers. Consequently, this may have affected the time devoted to family life and engagement in physical activity (PA). As the lockdown impacted people’s working patterns and amount of time spent at home, it potentially allowed for greater time to undertake physical activity. However, gyms and public sport centres were closed, so all exercise became self-motivated without the normal physical and social support of a structured training environment. This was the first time since the Second World War that a mass change in lifestyle had been enforced on the UK population and curfew measures have been shown to affect several aspects of lifestyle [3].

The UK Government recommends that adults aged 19–64 years should undertake 150 min of moderate physical activity or 75 min of vigorous physical activity per week [4]. Walking guidelines by Public Health England [5] recommend 30 min a day moderate intensity physical activity, to include walking, 5 days a week (or 150 min over a week) or 10 min of brisk walking per day for 7 days per week).

Physical inactivity has been defined as less than 30 min of physical activity a week [5]. Sedentary behaviour as defined by the World Health Organisation (2020) refers to behaviours in which energy expenditure is very low and sitting or lying is the dominant mode of posture [6]. Both physical inactivity and sedentary behaviour contribute to cardiovascular disease [7] as well as increasing the risk of obesity, type 2 diabetes and stroke with inactivity contributing to one in six deaths in the UK, the same number as smoking [8]. 

However, the Health Survey for England [9] showed that in 2016, only 66% of men and 58% of women aged 18 and over met the guidelines for aerobic activity. According to Public Health England (2017), 41% of adults aged 40–60 years in England walk less than 10 min continuously each month at a brisk pace and 19.7% of adults aged 40–60 years classed as inactive [5]. 

Although there are no defined guidelines on what should be considered the maximum amount of sedentary behaviour, the one sedentary behaviour considered a health threat is sitting [10]. From the epidemiological evidence, it is becoming clear that the associations of sitting time with all-cause mortality (ACM) and cardiovascular disease (CVD) mortality are often seen to be dependent on moderate to vigorous physical activity levels [11].

This survey aims to report whether there were changes in physical activity, sitting time and eating habits in the UK during the first lockdown of the COVID-19 outbreak as well as reporting perceived barriers to undertaking physical activity. Changes in lifestyle behaviours during the lockdown period may assist in predicting the impact of flexible and home working and whether this might be a lifestyle change that could improve the health of the population in future years and may determine in relation to physical activity, sitting time and physical inactivity. Arguably, more time for physical activity may be available to an individual working from home, but more time may also be spent sitting; therefore, detriments to health will also be considered. 

Subsequently, this research sets out to determine whether working from home impacts lifestyle, in particular frequency of physical activity, amount of physical inactivity and sitting time, along with eating habits. This may help to shape future working patterns in the UK.

## 2. Methods

### 2.1. Survey Details

This study was a descriptive analysis of the effect of lockdown during the SARS-CoV-2 pandemic in the UK on lifestyle. A 48-item online survey was created using Qualtrics (Qualtrics^XM^, Provo, UT, USA) following the NHS Live Well Survey [12], and the International Physical Activity Questionnaire (IPAQ) [13] and adapted with additional questions by the investigators). Some questions were displayed based on skip logic, so many participants responded to fewer than 48 questions. The average time taken to complete the survey was 51 min, with a range from 5 min to 5 days (after 5 days the questionnaire automatically timed out). When removing the outliers (deemed as those who did not complete the survey, taken in less than 8 min and those who left the survey open and returned some hours later, taken more than 60 min) the average time taken to complete the survey was 16 min. 

The survey was distributed via email correspondence through students and staff at the University of Hertfordshire and their associates and advertised on social media for a convenience sample of adults aged 18 years and above. Although this sampling may generate a sample that is skewed towards university staff and students, these participants represent those to whom staying at home during a lockdown will have the greatest impact.

Ethical approval was obtained through the University of Hertfordshire’s Health, Science, Engineering and Technology Ethics Committee with Delegated Authority, (protocol number LMS/SF/UH/04142) and no identifying data was collected. Consent was given by participation and completing the survey. The survey was live from 29 April to 13 May 2020 which was week five to seven of the UK lockdown, these were the weeks during the stay-at-home phase (phase 1) of the first UK lockdown. During this time people were told to stay inside their homes with limited exceptions for shopping or once a day for exercise such as running walking or cycling and for medical needs including providing care for another or to go to work. Gym and group exercise classes were not allowed during this time. The survey was retired at the ‘stay alert stage’ which included the phased re-opening of schools, non-essential retail, and more social and family contact.

The questionnaire evaluated the self-reported amount and type of physical activity, sitting time, perceived barriers to undertaking physical activity with regards to tiredness and time, weight change and eating habits for before and during lockdown. 

### 2.2. Scales and Description of Frequencies

The frequency of participation in physical activity was ordered such that never = not at all, rarely = once a month, sometimes = 1–2 times a month, often = 1–2 times a week and frequently = greater than twice a week.

Physical inactivity is defined as participating in less than 30 min of moderate intensity physical activity per week as defined by Public Heath England (2017) [14]. 

### 2.3. Data Analysis

Data were analysed using SPSS (version 26, IBM, New York, NY, USA), Excel (Microsoft Office 365, Microsoft, Irvine, CA, USA) and Social Science Statistics and Descriptive statistics (https://www.socscistatistics.com) (accessed on 26 September 2020). A contingency table analysis using Pearson’s-Chi squared or Fisher’s exact test, where appropriate, were used to determine whether a statistically significant difference was observed pre to during lockdown. All statistical tests were considered statistically significant at *p* ≤ 0.05.

## 3. Results

### 3.1. Descriptive Statistics

#### 3.1.1. Participants

A total of 818 participants completed the study (Table 1).

#### 3.1.2. Self-Reported Body Mass and Eating Habits

After 4 weeks of lockdown, self-reported body mass change showed an increase in weight in 32% of the cohort, with the majority (51%) staying the same and 17% reporting a decrease. Changes in food intake showed that 39.1% of the participants were eating more during the lockdown period compared to before, 15.3% were eating less and 45.6% were eating about the same. Participants also reported an increase in the number of portions of ‘bad’ or unhealthy snacks, high in fat, sugars or salt being eaten daily. An intake of greater than five portions per week increased from 269 to 384 participants, a 30% increase.

#### 3.1.3. Changes in Exercise Frequency during Lockdown for Gym and Exercise Class Participation 

**Figure 1:**
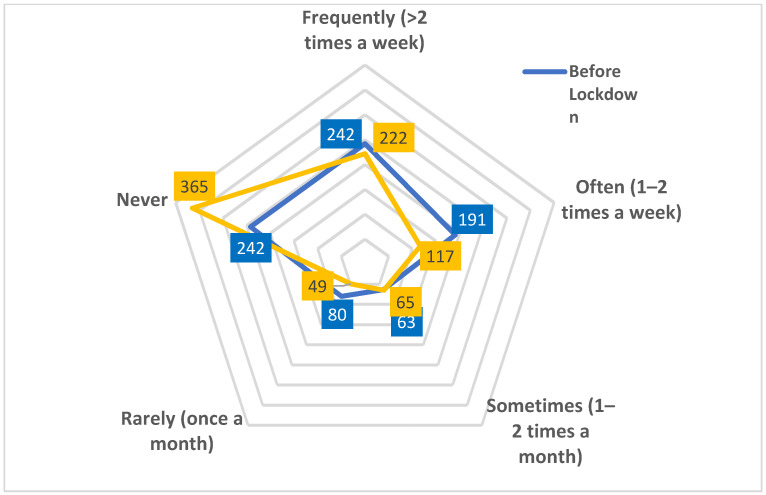
Frequency of using gym or exercise classes before and during lockdown, shown by number of participants for each category.

Of the 818 participants included in this study, 185 (22.6%) never went to a gym or undertook an exercise class (including on-line/virtual classes) either before or during lockdown. Of the 242 participants who went to a gym or undertook an exercise class frequently (>2 times a week) before lockdown (Figure 1), 120 (49.6%) continued this during lockdown, whilst gyms and group exercise classes were closed but home workouts and online classes continued. Sixty-four participants (26.4%) of those who frequently (>2 times per week) attended the gym or an exercise class before lockdown changed to never attending the gym or an exercise class during lockdown. Fifteen of the 242 participants (6.2%) who had never gone to the gym or undertaken an exercise class changed to frequently participating. 

From pre-lockdown to during lockdown, the total number of participants who never went to the gym or did exercise classes increased from 242 to 365 (50.8%). Those who went to the gym or took classes sometimes, often, or frequently before lockdown reduced from 496 to 404 during lockdown, (18.6% decrease). The relationship between these variables was significant, *X*^2^ (1, *N* = 818) 33.7, *p* < 0.001.

This data shows a large proportion of the sample that never went to a gym or undertook an exercise class before lockdown continued this behaviour during lockdown. Changes in exercise patterns during lockdown showed half of those frequently exercising before lockdown continued to do so; however, a notable proportion of this sub-sample moved from frequent use of gyms and exercise classes to never exercising (26.5%). Far less participants increased their participation in gym and exercise classes from before lockdown to during.

### 3.2. Exercise Modes

#### Changes in Outdoor Running and Cycling

From a total of 790 (28 non-responders) participants, 300 participants never cycled or ran before or during lockdown, 420 undertook outdoor running or cycling at least once a month before lockdown increasing to 490 during lockdown, a 17% increase. Moreover, there was a 38% increase in the number of participants (147 to 203) reporting to exercise more than twice a week (frequently), and a reduction in those who exercised less than once a month from before to during lockdown (559 to 478). This suggests a large change to exercise patterns during lockdown, with more people exercising at least once per month. A chi-square test of independence was performed to examine the relationship between those who exercised frequently before and during lockdown compared to those who exercise sometimes or less. The relationship between these variables was significant, *X*^2^ (1, *N* = 790) = 14.84, *p* = 0.00012.

### 3.3. Walking Behaviour

In response to how many times per week participants walked for a minimum of 30 min at a time, 26 participants never undertook a walk lasting ≥30 min either before or during lockdown. The number who undertook a walk of ≥30 min three or more times a week increased by 70% from 289 to 492 participants from before to during lockdown. Of the 289 participants who exercised ≥30 min two or more times a week before lockdown 32 (11%) reduced to less than once a week during lockdown.

The number of participants walking 150 min per week increased during lockdown resulting in an additional 11.4% of participants meeting the UK Government guidelines, [4] for moderate-intensity activity (Table 2) but with the majority (76%) still not achieving this through walking. However, with regards to 10-min daily walks there was a 23% increase in participants walking 10 min or more, seven days a week during lockdown compared to before (Table 2).

### 3.4. Inactivity

Assuming that no other exercise was taking place 8.6% (69 participants from 801) would be classified as sedentary from this data. For those classified as physically inactive (less than 30 min walking per week, 307 participants were classified as inactive before lockdown reducing to 267 during lockdown (13% reduction). 

### 3.5. Sitting Time

**Figure 2:**
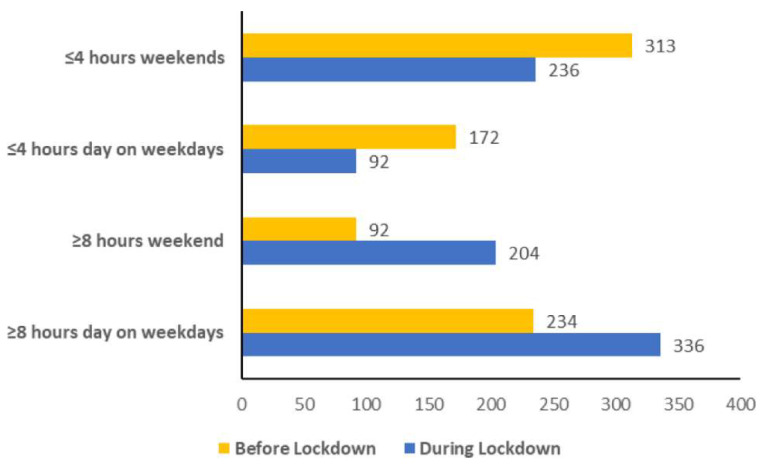
Number of participants sitting for ≤4 and ≥8 h per day on weekdays and weekends before and during lockdown.

Increases in percentage change from before to during lockdown for sitting time of ≥8 h per day could be seen on both weekdays (44%) and weekends (122%) (Figure 2). Those sitting for ≤4 h per day, percentage change from before to during lockdown reduced during weekends (−25%) and weekdays (−47%), indicating a large increase in those sitting for ≥8 h per day and a noticeable shift in sitting patterns during weekends (Figure 2).

### 3.6. Barriers to Physical Activity

Time was a barrier to exercise for 640 participants before lockdown who described not having enough time to exercise either sometimes, often, or most of the time. However, during lockdown this reduced by 60% to 240 participants. The relationship between these variables was significant, *X^2^* (1, *N* = 818) 367.5, *p* < 0.00001, showing that participants who stated lack of time to take part in physical activity had significantly decreased during lockdown. 

Before lockdown, 578 participants reported not feeling like undertaking physical activity due to tiredness either sometimes, often, or most of the time. During lockdown this decreased by 16% to 487 participants. These changes in barriers to physical activity concur with the increases shown in walking, running, and cycling activity.

## 4. Discussion

This study surveyed a cohort of the UK adult population to determine changes in physical activity and eating patterns during the first national lockdown against pre-lockdown measures. The study aimed to determine whether working from home and flexible working impacts lifestyle, in particular frequency and amount of physical activity, sitting time and food intake. The aims of the study were met with the following results.

### 4.1. Changes in Physical Activity during Lockdown

#### 4.1.1. Frequency of Gym and Exercise Class Participation

From Figure 1 it can be seen that there was an increase of 50.8% pre to during lockdown for those who never exercised at the gym or undertook an exercise class (either online or live) and a decrease of 53.3% during lockdown for those attending >2 times per week before lockdown to never during lockdown showing that many frequent exercisers did not engage with online and home workouts. These decreases in exercise suggests that those not meeting the minimum physical activity guidelines set out by the World Health Organisation [6] increased during lockdown. This was partly due to the gyms and health clubs being closed but the option of home and online workouts were freely available, although uptake appears low and does not appear to have positively impacted time spent exercising at home. Only 1.83% of the participants changed from never going to the gym or undertaking an exercise class before lockdown to frequently (>2 times per week) undertaking a gym or exercise class activity (at-home or on-line) during lockdown, suggesting that uptake of online workouts was not widespread.

The ramifications of such a finding are significant given that the risk of cancer, diabetes, osteoporosis, cardiovascular disease and poor mental health amongst individuals that never exercise are markedly elevated [15]. Furthermore, the risk of mortality and experiencing severe symptoms from COVID-19 itself is also likely increased, particularly in individuals with low physical activity levels [16].

#### 4.1.2. Moderate Exercise and Walking

This study found that there was a 70% increase in those walking at least 30 min continuously 3 or more times a week during lockdown, which although slightly below the minimum recommendation of 150 min per week is still a positive increase in the amount of PA undertaken by the participants and reduced sedentary behaviours as recommended by the WHO [6]. Current WHO guidelines on PA acknowledge that even a small amount of physical activity is better than none [6].

Of the 73 participants who never walked before lockdown, 26 continued to never walk during lockdown, however 21 of this cohort did start to walk frequently (>2 times per week) during lockdown. The curvilinear dose-response relationship between physical activity and health benefits suggests the greatest proportional benefits occur when progressing from inactive or very low levels of physical activity to moderate levels, even if they are below the minimum threshold recommended by guidelines [4] as seen above. 

The increase in physical activity in the form of walking concurs with the findings of a study in Italy [3]. In a subsample of the Italian population physical activity frequency was significantly increased during lockdown compared to pre-lockdown, although there was no change in frequency in those that were not exercising before lockdown. Our results also align with data from an international online survey from four different continents which reported a significant reduction in total time spent doing physical activity, during COVID-19 lockdown compared to before lockdown [17]. 

In the current study, less than 25% of participants met the minimum guidelines of 150 min of moderate intensity physical activity per week either before or during lockdown. In comparison, the UK national estimate of people meeting the minimum guideline of 150 min of moderate intensity exercise as of November 2019 was 67% for adults aged 19 and over [18]. It should be noted that our sample over-represents females who tend to have lower physical activity levels compared to males in the UK [18], which may explain some of the discrepancy between our sample and national estimates. Furthermore, only walking was used as an estimate of moderate exercise as all other forms of moderate exercise (such as doubles tennis and aqua aerobics) were not available during the lockdown. 

National guidelines, recommend including walking, 5 days a week (or 150 min over a week) or 10 min per day for 7 days per week [5]. The frequency of undertaking a minimum 10 min walk 7 days per week increased from 30% before lockdown to 37% during lockdown (Table 2), indicating more participants were able to be physically active each day. 

#### 4.1.3. Outdoor Running and Cycling

Outdoor exercise patterns changed during lockdown compared to pre-lockdown, as the number of participants exercising by running or cycling increased, with individuals exercising >2 times per week showing a 38% increase. Those exercising less than once a month reduced from 559 to 478 participants, resulting in an increase in those exercising once a month or more. These changes were most likely brought about by the enforced rules that people could only go outside to exercise once a day therefore participants who wished to continue exercising decided to optimise this opportunity by running and cycling. Conversely, those who had no interest in running or cycling before lockdown continued to have no interest during with the majority of participants (300) never cycling or running either before or during lockdown. This is not to say that these participants did not undertake more walking as discussed previously.

### 4.2. Physical Inactivity 

Physical inactivity, defined as <30 min moderate intensity activity per week in the UK, is a risk factor for developing disease and can hinder the ability to resist viral infection [16]. The rate of physical inactivity during lockdown likely increased across the world, with global average step count decreasing up to 38% on the same period last year according to data from the 30 million global users of wearable technology company FitBit [19]. Our data showed a 13% decline in individuals performing <30 min exercise per week during lockdown compared to pre-lockdown amongst those that were classed as physically inactive before lockdown. Of those that were physically active (>30 min per week) before lockdown, 36% became physically inactive during lockdown and of the 289 participants who exercise >30 min 2 or more times a week before lockdown, 32 (11%) reduced to less than once a week during lockdown. This is a surprising finding of the study where lockdown had caused people to work from home and were able to self-regulate the amount of time spent walking. However, as mentioned above the overall data showed an increase of 70% for those who walked >30 min per week, during lockdown, which would give health benefits to those individuals. However, despite time spent walking 30 min per week increasing, there was also an increase in the duration of sitting time in our sample during lockdown compared to pre-lockdown. 

During weekdays where people are often sedentary due to office work the number of people sitting for 8 h or more a day rose by 43.6% from 234 to 336 participants. However, at weekends the number of people sitting for more than 8 h a day increased by 121% from 92 people to 204 people. To compound the issue of people being sedentary during lockdown, it was also seen that those sitting for less than 4 h per day decreased during lockdown during the week and at weekends by 46.5% and 25.6%, respectively. Similarly, in a Belgian population, sitting time increased during COVID-19 lockdown [20]. The importance of this requires highlighting since increased sitting time (>4 h per day) has been shown to be a risk factor for all-cause mortality, independent of physical activity levels [21]. The estimated national average for sitting time in the UK is 9 h per day, with premature mortality risk increasing for those sitting >7 h per day and an additional 5% greater risk for every hour thereafter [22]. In this study 41.1% of total participants sat for more than 8 h a day on weekdays. Prolonged sitting increases health risk even in those meeting minimum physical activity guidelines [4]. Breaking up prolonged periods of sitting time, even with standing or light activity, has been associated with improved cardio-metabolic health [23]. Evidence suggests 2–3 min of light activity every 20–30 min over several hours improves cardio-metabolic health [24], and would be recommended generally but especially during periods of confinement where sitting time is likely to be increased. Increases in 10 min walks on 7 days a week seen in this study and discussed above would help to counteract this sedentary behaviour.

### 4.3. Body Mass and Eating Habit Changes

Thirty-two percent of the participants in this study self-reported an increase in body mass after the first 4 weeks of lockdown. This gain in weight may be attributed to changes in eating habits but also changes in physical activity levels and sitting time. The majority of participants (51%) reported no change in weight with 17% reporting a decrease. These figures correspond to the changes to eating habits, with 39.1% of the participants self-reporting an increase in food intake and only 15.3% eating less. Further to this there was a 30% increase in those eating five or more portions of ‘bad’ or unhealthy food each week. An increase in body mass would be expected with this increase in food intake and unhealthy food, despite the increased walking times. Walking does not tend to utilise high amounts of calories and is negated by the increase in reported sitting time and concomitant decrease in exercise frequency, therefore an increase in body mass would be expected.

### 4.4. Barriers to Physical Activity

Lack of time has been suggested as a barrier to physical activity and this is further supported by our data showing individuals cited having more time to exercise increasing by 60% during lockdown amongst those who said they felt like not exercising ‘sometimes’, ‘often’ or ‘most of the time’ before lockdown. Another encouraging aspect of the work from home directive during lockdown, was that there was a 16% decrease in those feeling too tired to exercise. Feeling less tired, along with extra time to exercise, whilst working from home, may help more of the population to achieve recommended physical activity guidelines [4]. It is an aspect of the lockdown which needs to be considered for future working patterns when policy on returning to work is being considered.

This has important implications for promoting better health since the UK Chief Medical Officer recommends daily physical activity [4], suggesting employees and policymakers should reconsider working patterns to enable more time for physical activity to be performed daily.

### 4.5. Considerations to Working from Home and Future Lockdowns 

This study shows that increases in frequency of daily walks and amount of weekly walking times and decreases in barriers to exercise such as time and tiredness occurred during the lockdown. We recommend that these results should be considered and recognised in future decision making for people to work from home and have flexible working patterns as they show that people have more time and feel less tired to undertake PA. 

Although, other sedentary behaviour and changes in eating patterns may have been negatively impacted by the lockdown, it must be considered that during this time the people of the UK did not have access to sporting or gym facilities and were having to stay indoors, which would not be the case outside of a lockdown. 

We recommend practical advice for individuals in case of future lockdowns leading to confinement and limited social interaction, to follow recommendations for physical activity with respect to any guidelines set out by respective authorities such as maintaining social distancing. Individuals should undertake physical activity as frequently as possible, preferably daily, to maintain good health during any lockdown periods [16] and in everyday life. Utilisation of technology such as online classes, wearable sensors and mobile applications can provide social interaction and motivation to become more physically active in a socially distanced environment. However, when society opens up fully again, the additional benefits of time and lack of tiredness, as seen during this lockdown may assist those working from home to undertake more PA.

### 4.6. Strengths/Limitations and Future Work

Results from our study must be interpreted with respect to limitations. Individuals were required to self-report thus potentially introducing some bias into the findings. Our dataset also over-represented females and the white ethnicity which may have influenced responses to certain questions. Online surveys are not always representative of the wider population and our sample size was relatively small for an online survey and as advertised through the University, as well as on social media, may have attracted more students than the general population, full descriptive and demographic information can be found in Table 1. However, given restrictions on social interaction due to lockdowns, online surveys were utilised similar to other studies during the COVID-19 pandemic [3,17]. A strength of our study was determining responses during the peak of the COVID-19 lockdown, however future work should consider determining the long-term changes to lifestyle patterns following the COVID-19 lockdown and whether patterns are influenced by future lockdowns. Further baseline data was self-reported along with week 5 data, which was a limitation of data collection during the COVID-19 lockdowns.

Future work may also consider sub-group analysis based on independent variables (age, sex, ethnicity, household income, physical activity levels) on lifestyle patterns to minimise the imbalances in sub-samples of the population as well as looking at the impact on family life and time spent exercising with children. Lastly, a qualitative analysis of reasons as to why lifestyle patterns were or were not changed during lockdown would be useful to inform public health and policy.

## 5. Conclusions

During lockdown time spent walking, running and cycling increased; however, the majority of people still did not meet the Government guidelines for physical activity. Less people cited lack of time and feelings of tiredness as barriers to PA during lockdown, which should be considered in future flexible working and working from home policies. Sitting time increased both during weekdays and weekend days along with food intake during lockdown. Gains are especially significant for those currently doing the lowest levels of activity (fewer than 30 min per week), as the improvements in health per additional minutes of physical activity will be proportionately greater, and this was the area that most improvements were found.

Regular PA and breaking up prolonged periods of sitting time are important in the prevention of severe health complications during lockdowns and during everyday life and to meet the Government and WHO guidelines for PA. However, more time and less tiredness may encourage people to undertake more PA when working from home.

## Figures and Tables

**Table 1:** Participant Characteristics (*n* = 818).

Personal Details	Age (Years) (Mean ± SD)	BMI (kg.m^−2^) (Mean ± SD)	Males (Total Number)	Females (Total Number)				
	47 ± 13	26 ± 5	177	641				
**Education**	No Schooling	GCSE or equivalent	A level or BTEC or Apprenticeship or equivalent	Bachelor’s degree	**Master’s degree**	**Doctorate**		
**Total number**	3	82	203	279	160	91		
**Employment**	**Key Workers**	**Furloughed**	**Working from home**	**Working outside from home**	**Retired**	**Unemployed**	**Carer for family member**	
**Total number**	188	113	483	98	37	83	4	
**Income**	**<£5200**	**£5200 to £15,999**	**£15,600 to £25,999**	**£26,000 to £36,399**	**£36,400 to £51,999**	**£52,000 to £77,999**	**£78,000 and above**	**Prefer not to say**
**Total number**	3	28	49	83	147	192	217	99
**Ethnicity**	**White**	**Asian**	**Black**	**Chinese**	**Mixed**	**Other/Prefer not to say**		
**Total number**	754	26	6	3	12	10		

**Table 2:** Total number of participants and percentage change from before to during lockdown of those completing <150 or ≥150 min of moderate-intensity activity per week and walks lasting ≥10 min, seven days a week.

Walking Time	Before Lockdown	during Lockdown	Before to during % Change
**<150 min per week**	639	621	−2.8
**≥150 min per week**	176	194	11.4
**10-min Daily Walks**	244	300	23

## Data Availability

Data underpinning this publication are available at https://doi.org/10.18745/ds.25047.

## References

[1] World Health Organisation (2020). Coronavirus Disease (COVID-19) Pandemic. https://www.euro.who.int/en/health-topics/health-emergencies/international-health-regulations/news/news/2020/2/2019-ncov-outbreak-is-an-emergency-of-international-concern.

[2] GOVUK (2020). UK Government Prime Minister’s Statement on Coronavirus (COVID-19): 23 March 2020. https://www.gov.uk/government/speeches/pm-address-to-the-nation-on-coronavirus-23-march-2020.

[3] Di Renzo L., Gualtieri P., Pivari F., Soldati L., Attinà A., Cinelli G., Cinelli G., Leggeri C., Caparello G., Barrea L. (2020). Eating habits and lifestyle changes during COVID-19 lockdown: An Italian survey. J. Transl. Med..

[4] Davies D.S.C., Atherton F., McBride M., Calderwood C. (2019). UK Chief Medical Officers’ Physical Activity Guidelines.

[5] Public Health England (2017). 10 Minutes Brisk Walking Each Day in Mid-Life for Health Benefits and towards Achieving Physical Activity Recommendations: Evidence Summary. https://assets.publishing.service.gov.uk/government/uploads/system/uploads/attachment_data/file/639030/Health_benefits_of_10_mins_brisk_walking_evidence_summary.pdf.

[6] Bull F.C., Al-Ansari S.S., Biddle S., Borodulin K., Buman M.P., Cardon G., Carty C., Chaput J.P., Chastin S., Chou R. (2020). World Health Organization 2020 guidelines on physical activity and sedentary behaviour. Br. J. Sport. Med..

[7] British Heart Foundation [Internet] (2017). Physical Inactivity and Sedentary Behaviour Report. British Heart Foundation. https://www.bhf.org.uk/informationsupport/publications/statistics/physical-inactivity-report-2017.

[8] Public Heath England (2014). Everybody Active, Every Day.

[9] Scholes S. (2017). Health Survey for England 2016 Physical Activity in Adults. http://healthsurvey.hscic.gov.uk/media/63730/HSE16-Adult-phy-act.pdf.

[10] Stamatakis E., Ekelund U., Ding D., Hamer M., Bauman A.E., Lee I.-M. (2019). Is the time right for quantitative public health guidelines on sitting? A narrative review of sedentary behaviour research paradigms and findings. Br. J. Sport. Med..

[11] Petersen C.B., Bauman A., Grønbæk M., Helge J.W., Thygesen L.C., Tolstrup J.S. (2014). Total sitting time and risk of myocardial infarction, coronary heart disease and all-cause mortality in a prospective cohort of Danish adults. Int. J. Behav. Nutr. Phys. Act..

[12] NHS (2019). NHS Live Well. https://www.nhs.uk/live-well/sleep-and-tiredness/how-to-get-to-sleep/.

[13] Hagströmer M., Oja P., Sjöström M. (2006). The International Physical Activity Questionnaire (IPAQ): A study of concurrent and construct validity. Public Health Nutr..

[14] Public Heath England (2017). BRISK Walking and Physical Inactivity in 40 to 60 Year Olds. https://documentcloud.adobe.com/link/review?uri=urn:aaid:scds:US:d504adec-351a-4d9d-8e50-2f4668601c0c.

[15] World Health Organisation (2002). Physical Inactivity a Leading Cause of Disease and Disability, Warns WHO. https://www.who.int/news/item/04-04-2002-physical-inactivity-a-leading-cause-of-disease-and-disability-warns-who.

[16] Woods J.A., Hutchinson N.T., Powers S.K., Roberts W.O., Gomez-Cabrera M.C., Radak Z., Berkes I., Boros A., Boldogh I., Leeuwenburgh C. (2020). The COVID-19 pandemic and physical activity. Sport. Med. Health Sci..

[17] Ammar A., Brach M., Trabelsi K., Chtourou H., Boukhris O., Masmoudi L., Bouaziz B., Bentlage E., How D., Ahmed M. (2020). Effects of COVID-19 Home Confinement on Eating Behaviour and Physical Activity: Re1sults of the ECLB-COVID19 International Online Survey. Nutrients.

[18] NHS (2020). Statistics on Obesity, Physical Activity and Diet, England, 2020. https://digital.nhs.uk/data-and-information/publications/statistical/statistics-on-obesity-physical-activity-and-diet/england-2020.

[19] FitBit (2020). FITBIT News: The Impact of Coronavirus on Global Activity. https://blog.fitbit.com/covid-19-global-activity/.

[20] Constandt B., Thibaut E., De Bosscher V., Scheerder J., Ricour M., Willem A. (2020). Exercising in Times of Lockdown: An Analysis of the Impact of COVID-19 on Levels and Patterns of Exercise among Adults in Belgium. Int. J. Environ. Res. Public Health.

[21] Van Der Ploeg H.P., Chey T., Korda R.J., Banks E., Bauman A. (2012). Sitting time and all-cause mortality risk in 222 497 Australian adults. Arch. Intern. Med..

[22] Buckley J.P., Hedge A., Yates T., Copeland R.J., Loosemore M., Hamer M., Bradley G., Dunstan D.W. (2015). The sedentary office: A growing case for change towards better health and productivity. Expert statement commissioned by Public Health England and the Active Working Community Interest Company. Br. J. Sport. Med..

[23] Benatti A., Ried-Larsen M. (2015). The Effects of Breaking up Prolonged Sitting Time: A Review of Experimental Studies. Med. Sci. Sports Exerc..

[24] Chastin S.F.M., Egerton T., Leask C., Stamatakis E. (2015). Meta-analysis of the relationship between breaks in sedentary behavior and cardiometabolic health. Obesity.

